# From tool to scaffold: structured human–AI collaboration and its effects on academic writing and digital critical thinking among Saudi EFL learners

**DOI:** 10.3389/fpsyg.2026.1830103

**Published:** 2026-05-26

**Authors:** Omar Abdullah Omar Alshehri, Ali Lamouchi, Mohamed Sayed Abdellatif, Mashael Nasser Ayed Al-Dosari, Mohamed Mekheimer, Mohamed Ali Nemt-allah

**Affiliations:** 1 Department of Library and Information Science, College of Education and Human Development, University of Bisha, Bisha, Saudi Arabia; 2 Department of English Language and Literature, College of Science and Humanities, Prince Sattam Bin Abdulaziz University, Al-Kharj, Saudi Arabia; 3 Department of Psychology, College of Education in Al-Kharj, Prince Sattam Bin Abdulaziz University, Al-Kharj, Saudi Arabia; 4 Department of Educational Sciences, College of Education in Al-Kharj, Prince Sattam Bin Abdulaziz University, Al-Kharj, Saudi Arabia; 5 Department of Teaching English to Speakers of Other Languages (TESOL), Faculty of Education, Beni-Suef University, Beni-Suef City, Egypt; 6 Department of Educational Psychology and Statistics, Faculty of Education, Al-Azhar University, Dakahlia, Egypt

**Keywords:** AI literacy, AI-supported writing, digital critical thinking, EFL academic writing, ethical regulation, generative AI, human–AI collaboration, Saudi higher education

## Abstract

This mixed-methods quasi-experimental study examined whether pedagogically structured, AI-supported writing instruction is associated with improvements in EFL academic writing performance and digital critical thinking/AI literacy among Saudi university students. Fifty-three undergraduates were assigned by intact classes to an experimental group (*n* = 31) or a control group (*n* = 22). Over 10–11 weeks, the experimental group practiced guided human–AI collaboration workflows—encompassing problem framing and prompt design, iterative drafting, revision cycles, verification of claims and citations, and responsible-use regulation—using a generative AI assistant and a language-feedback tool, while the control group completed the same syllabus without systematic AI integration. Both groups completed parallel pretest/posttest writing tasks (0–20 scale) and the Digital Critical Thinking Scale (DCTS; 20 items; 20–100; *α* = 0.97 pretest, α = 0.99 posttest). At posttest, the experimental group scored significantly higher on writing proficiency (*M* = 15.23 vs. 11.91; large effect) and DCTS total (*M* = 89.84 vs. 56.18; very large effect). Writing proficiency and DCTS were strongly associated at both posttest and in gain scores. Thematic analysis of experimental-group reflections revealed that students employed AI primarily as a planning and revision scaffold, while simultaneously enacting verification routines and articulating ethical self-regulation strategies. This study contributes context-specific empirical evidence demonstrating that agency-preserving, critically guided AI integration can simultaneously advance writing quality and digital critical thinking in EFL higher education contexts where such paired outcome evidence remains scarce. However, results should be interpreted as preliminary and institution-specific, given the modest sample, intact-class assignment, and single-institution context, which limit generalizability to other EFL populations and settings.

## 1 Introduction

Artificial intelligence (AI)—and generative AI in particular—is rapidly reshaping academic writing in higher education (Ansari and Qamari, 2025; Garzón et al., 2025; Jaboob et al., 2025). Its significance extends far beyond automating routine language editing tasks; it fundamentally alters the conditions under which learners plan, generate, evaluate, and revise ideas, while simultaneously introducing new demands for critical judgment, source verification, accurate attribution, and responsible authorship. Academic writing is therefore an especially productive and informative site for investigating AI’s educational impact, because effective writing requires sustained control over purpose, evidence, organization, and voice, alongside ethically grounded decisions about sources, originality, and acceptable forms of assistance (Deep and Chen, 2025; Habib et al., 2024; Urmeneta and Romero, 2025).

Despite rapid growth in AI-supported writing research, three empirical gaps remain particularly salient for Saudi university EFL writing contexts. First, although numerous studies report writing-related benefits from AI tool use, most rely on perception-based measures, correlational designs, or brief tool exposure, leaving unresolved whether sustained, pedagogically structured AI integration—compared against a matched control condition—produces meaningful writing gains beyond those achievable through conventional instruction alone (Al-Dossary, 2024; Al-Harbi and Al-Ahdal, 2025). Second, while “critical thinking” and “digital literacy” are frequently invoked as desired outcomes of AI integration, the literature is inconsistent in how these constructs are operationalized: few studies measure digital critical thinking as a multi-dimensional, assessable construct directly relevant to generative AI risks—specifically, hallucination detection, credibility evaluation, verification behavior, and responsible-use regulation—leaving a gap between conceptual claims and empirical measurement (Deep and Chen, 2025; Zakaria et al., 2025). Third, existing Saudi and regional frameworks offer valuable policy and conceptual guidance aligned with Vision 2030, yet they rarely provide empirically tested instructional workflows that preserve student agency while making verification and ethical regulation explicit, measurable components of writing pedagogy—a gap that limits the field’s ability to identify which instructional features are necessary for beneficial outcomes (Al-Fraidan, 2024; Elmahdi et al., 2025).

The present study addresses these gaps by implementing a quasi-experimental mixed-methods pretest–posttest design comparing structured AI-supported writing instruction with conventional instruction, measuring changes in writing proficiency and digital critical thinking using validated pre/post assessments, and triangulating quantitative outcomes with learners’ descriptions of verification routines and responsible-use practices. In doing so, this study moves beyond descriptive or perception-based accounts to provide context-specific causal-inferential evidence about how pedagogically governed AI use can simultaneously develop writing quality and digital critical thinking—two outcomes that existing literature treats largely in isolation—within an underrepresented Saudi EFL higher education context. Specifically, the study was guided by the following research questions:

- RQ1: To what extent does pedagogically structured AI-supported writing instruction improve Saudi university EFL students’ academic writing performance compared with traditional instruction?
- RQ2: To what extent does such instruction improve students’ digital critical thinking and AI literacy?
- RQ3: What is the relationship between writing performance and digital critical thinking at pre-test, post-test, and in gain scores?
- RQ4: How do students describe their human–AI writing workflows regarding idea development, revision, verification, and responsible-use regulation?

## 2 Literature review

### 2.1 AI-supported writing in EFL contexts

Recent scholarship has increasingly highlighted that the educational value of generative AI in writing contexts is shaped less by tool availability than by the quality of human–AI interaction fostered through deliberate instructional design (Aljuaid, 2024; Deep and Chen, 2025). Systematic reviews converge on two key points: first, AI tools can support improvements in linguistic accuracy, textual coherence, and genre awareness through immediate, individualized feedback; and second, these benefits are contingent on how AI is embedded within instruction rather than on tool availability alone. The strongest effects emerge when AI is integrated into process-oriented pedagogies involving iterative drafting, structured revision cycles, and explicit guidance on evaluating and selectively adapting AI suggestions (Aljuaid, 2024; Han and Li, 2024; Khalifa and Albadawy, 2024). When students are guided to frame problems, design prompts, compare alternative drafts, interrogate outputs, and document decision rationales, AI can function as a “cognitive sparring partner” that accelerates ideation and revision while strengthening habits of evaluation and self-regulation (Al-Mahmud, 2023; Garzón et al., 2025; Liu et al., 2025). Conversely, when used without instructional scaffolding as a shortcut to finished text, AI can undermine independent composing, weaken engagement with primary sources, blur authorial boundaries, and introduce integrity risks particularly consequential in academic writing contexts (Al-Nofaie and Alwerthan, 2024; Al-Sofi, 2024; Deep and Chen, 2025).

The mechanisms through which AI supports writing improvement operate across several interrelated processes. First, AI tools provide immediate, individualized diagnostic feedback on linguistic accuracy, syntactic complexity, and genre-level organization—otherwise constrained by class size and instructor availability—enabling students to identify and repair surface and structural weaknesses across multiple drafting cycles (Chan et al., 2024; Mekheimer, 2025; Shen et al., 2023; Song and Song, 2023). Second, AI functions as an ideational scaffold during planning stages: by generating alternative outlines, proposing argumentative structures, and modeling genre conventions, AI expands learners’ rhetorical options—particularly valuable for those lacking sufficient exposure to academic writing schemas (Deep and Chen, 2025; Kim et al., 2024; Reza et al., 2025; Wang, 2024). Third, iterative AI-mediated revision cycles make the writing process more visible and deliberate—students compare AI-generated alternatives against their own drafts, evaluate competing formulations, and make explicit accept-or-reject decisions that strengthen metacognitive awareness of compositional choices (Chan et al., 2024; Hong et al., 2025; Nguyen et al., 2024). Fourth, lexical and register feedback supports vocabulary development by surfacing precise academic alternatives to informal word choices, improving clarity and disciplinary appropriateness (Deep and Chen, 2025; Mekheimer, 2025; Shen et al., 2023; Wang, 2024). Crucially, these mechanisms produce learning gains only when instruction structures students to critically engage with AI feedback rather than passively accept it—making human–AI interaction quality, rather than tool access alone, the proximate driver of writing improvement (Aljuaid, 2024; Arif et al., 2025; Deep and Chen, 2025).

Empirical work in Saudi and regional EFL settings has reported positive associations between AI tool integration and writing-related outcomes such as accuracy, organizational coherence, and learner confidence (Abduljawad, 2024; Alarifi et al., 2024; Alzahrani F. and Alotaibi H, 2024). Parallel studies have documented benefits across broader language skill areas—vocabulary acquisition, reading comprehension, and oral communication—in AI-mediated instructional environments (Alghamdi, 2024; Hashemifardnia and Kooti, 2025). However, much of this work shares notable methodological limitations: reliance on self-report instruments, single-group designs without control conditions, and short intervention periods that make it difficult to distinguish genuine skill development from familiarity effects or response bias (Alzahrani F. and Alotaibi H, 2024). Nevertheless, reported benefits vary considerably across studies, with inconsistencies attributable to differences in instructional design, assessment rigor, and intervention duration—limiting cross-study comparability and generalizability of positive findings (Al-Dossary, 2024; Al-Sofi, 2024).

### 2.2 Digital critical thinking and AI literacy

Digital critical thinking has emerged as a central conceptual concern in debates about AI integration in higher education. Students in AI-rich learning environments require capacities specific to algorithmically mediated information ecologies: interrogating digital information flows, evaluating AI-generated claims for accuracy and bias, identifying hallucinated or fabricated content, triangulating information with reliable scholarly sources, and making ethically grounded decisions about attribution and acceptable levels of AI assistance (Al-Zahrani and Alasmari, 2025; Deep and Chen, 2025; Faisal, 2024; Zakaria et al., 2025). Within this emerging scholarship, digital critical thinking overlaps substantially with AI literacy: the capacity to evaluate credibility, detect bias and hallucination, triangulate claims against reliable sources, and regulate ethical use (Liu et al., 2025; Zakaria et al., 2025).

Research in Saudi higher education suggests that stronger digital competence and critical awareness are associated with improved learning outcomes, enhanced digital well-being, and more sustainable technology engagement (Abou Hashish and Alnajjar, 2024; Ahmed and Ali, 2024; Alruwaili et al., 2025). AI-critical pedagogic approaches have proposed specific instructional strategies—counter-prompting, error detection tasks, and reflective questioning—to embed critical engagement with AI outputs as a habitual classroom practice (Ajani et al., 2025; Alqarni, 2024; Kassenkhan et al., 2025). Yet a persistent inconsistency exists between the theoretical centrality of digital critical thinking and its empirical treatment: most studies operationalize it through general scales not designed for AI-mediated contexts, leaving claims about AI fostering critical thinking largely aspirational rather than empirically grounded (Deep and Chen, 2025; Zakaria et al., 2025).

Several frameworks in Saudi EFL and broader regional higher education explicitly link AI integration to critical thinking development and culturally responsive pedagogy. Elmahdi et al. (2025) propose a culturally responsive approach to integrating critical thinking and technology in Saudi EFL classrooms, foregrounding AI-enhanced analytical tasks aligned with local values and Vision 2030 objectives. Other regional contributions similarly emphasize moving beyond basic digital literacy toward higher-order digital competencies—problem solving, ethical reasoning, and reflective judgment—through carefully designed AI-mediated learning experiences (Alshraah et al., 2024; Ilgun Dibek et al., 2025). Evidence from AI-enhanced courses in Saudi higher education suggests that carefully designed AI activities can strengthen analytical reasoning and data literacy when they require explicit justification and verification behaviors (Al-Fraidan, 2024; Alhathli, 2024).

### 2.3 Saudi higher education, Vision 2030, and AI integration

In Saudi Arabia, pedagogical questions surrounding AI intersect directly with Vision 2030 priorities emphasizing digital transformation, innovation, and human-capital development (Alotaibi and Alshehri, 2023; Al-Zahrani and Alasmari, 2025). Reviews focusing on Saudi higher education highlight both the promise and the institutional constraints of AI adoption: AI-based systems may enhance learning flexibility and higher-order skill development, but implementation outcomes are frequently moderated by infrastructure readiness, staff training, and unresolved ethical questions (Alotaibi and Alshehri, 2023). A systematic review focused specifically on ChatGPT in Saudi higher education argues that generative AI can support scaffolding and higher-order learning—provided that integration is appropriately structured and ethically bounded, with explicit institutional attention to plagiarism prevention, overreliance mitigation, and bias awareness (Faisal, 2024).

At the same time, perception-based research among Saudi students and faculty consistently highlights a fundamental ambivalence: AI tools are valued for their efficiency and linguistic support functions, yet they are simultaneously perceived as a threat to authentic learning, independent thinking, and ethical academic practice (Aladsani, 2024; Aldossary et al., 2024; Al-Smadi et al., 2024; Aluthman, 2024). Writing-focused research in Saudi contexts indicates that EFL learners commonly use AI for idea generation, initial drafting, and surface polishing, reporting improved clarity and correctness alongside mixed feelings regarding originality and authorial ownership (Al-Harbi and Al-Ahdal, 2025; Al-Sofi, 2024; Alyami et al., 2025). This tension has strengthened calls within the field for pedagogical models that position AI as a scaffold for higher-order learning and genuine intellectual development, rather than as a substitute for student authorship and cognitive engagement. Regional frameworks have emphasized culturally responsive and ethically aligned integration, arguing that AI-supported learning should respect local values and Vision 2030 priorities while actively promoting higher-order judgment over passive acceptance of machine-generated content (Alhathli, 2024; Elmahdi et al., 2025).

In Saudi and regional contexts, AI is increasingly positioned as a means to foster higher-order skills, but with a consistent conditional emphasis: benefits depend on whether instruction prompts students to justify, compare, verify, and revise AI-generated recommendations against credible external evidence (Al-Fraidan, 2024; Al-Housni et al., 2024). Systematic reviews of AI in education describe accelerating use of AI tools for adaptive learning, intelligent tutoring, automated assessment feedback, and writing support, with reported associations to enhanced learner engagement, higher feedback quality, and a broad range of cognitive outcomes (Garzón et al., 2025; Ilgun Dibek et al., 2025).

### 2.4 Theoretical framework

This study is grounded in an integrated theoretical framework combining constructivist and sociocultural accounts of mediated learning, digital critical thinking and AI literacy perspectives, and process-writing and self-regulation models. Constructivist theory treats learning as an active, iterative process in which students build knowledge through cycles of feedback, revision, and reflection—processes that align directly with AI-supported writing workflows (Al-Mahmud, 2023; Liu et al., 2025). Sociocultural theory extends this view by emphasizing that learning is mediated by cultural tools; generative AI can thus be conceptualized as a mediational means operating within the learner’s zone of proximal development, capable of proposing outlines, modeling genre conventions, and surfacing alternatives that students may not readily produce independently (Al-Harbi and Al-Ahdal, 2025; Hashemifardnia and Kooti, 2025). However, the central boundary condition is that learning benefits depend on how learners interact with mediation—when students engage with AI outputs by questioning, comparing drafts, rejecting or adapting suggestions, and justifying compositional choices, the interaction constitutes active mediated learning rather than passive text transfer (Garzón et al., 2025; Ilgun Dibek et al., 2025).

From a process-writing and self-regulation perspective, the educational value of AI depends on whether it makes the writing process more visible and subject to deliberate revision, rather than outsourcing final text production (Aljuaid, 2024; Al-Sofi, 2024; Deep and Chen, 2025). Synthesizing these perspectives, the study’s central theoretical claim is conditional: AI applications can act as mediational tools supporting both writing development and digital critical thinking only when instruction structures human–AI interaction to be critical, verificatory, and responsibility-oriented. This theoretical synthesis directly informs the instructional design and outcome measures adopted in the present study, as detailed in the following section.

## 3 Materials and methods

### 3.1 Research design

This study employed a convergent mixed-methods quasi-experimental pretest–posttest control-group design, supplemented by qualitative thematic analysis of learner reflections. The independent variable was instructional approach (AI-supported writing instruction versus traditional writing instruction without systematic AI integration). The primary dependent variables were writing proficiency and digital critical thinking/AI literacy. Quantitative and qualitative strands were integrated through triangulation and a joint display, enabling meta-inferences about both outcome patterns and the instructional processes most plausibly associated with those patterns. Three null hypotheses guided the statistical analyses:

*H1*: There is no statistically significant difference in academic writing performance between the experimental and control groups at posttest.

*H2*: There is no statistically significant difference in digital critical thinking/AI literacy scores between the experimental and control groups at posttest.

*H3*: There is no statistically significant relationship between academic writing performance and digital critical thinking/AI literacy scores at pretest, posttest, or in gain scores.

### 3.2 Participants and setting

Participants were 53 Saudi undergraduate EFL students enrolled in a required academic writing course at a Saudi public university. Two intact course sections were assigned to the experimental group (*n* = 31) and the control group (*n* = 22). Inclusion criteria were enrollment in the target course, attendance at both pretest and posttest measurement points, and provision of informed consent. Students missing either measurement point were excluded from the analytic sample. Baseline equivalence analyses confirmed no statistically significant between-group differences on pretest writing scores, pretest DCTS scores, or key background variables including gender, age, self-reported proficiency level, and prior AI use, indicating that observed post-intervention differences are unlikely to reflect substantial pre-existing group disparities.

### 3.3 Instruments

#### 3.3.1 Writing proficiency assessment

Writing proficiency was assessed through a timed academic essay task (45 min) scored using an analytic rubric comprising four dimensions: content, organization, language use, and mechanics. Scores across dimensions were summed to produce a total score ranging from 0 to 20. Parallel prompts were used for pretest and posttest, each requiring a 250–300-word essay on a comparable academic topic. Two trained raters independently scored all scripts, and the mean of the two ratings served as each student’s writing score.

Writing performance was evaluated using an analytic rubric comprising four weighted dimensions: content (0–8 points), which assessed the relevance, depth, and development of ideas, including argumentation quality, supporting evidence, and topic adherence; organization (0–5 points), which evaluated logical essay structure, clarity of introduction and conclusion, paragraph coherence, and transitional devices; language use (0–5 points), which examined grammatical accuracy, syntactic complexity, academic register, and vocabulary precision; and mechanics (0–2 points), which addressed spelling, punctuation, and formatting accuracy. Scores across all four dimensions were summed to produce a total writing proficiency score ranging from 0 to 20. Two trained raters independently scored all scripts blind to group assignment, with the mean of the two ratings serving as each student’s final writing score. Inter-rater reliability was established prior to formal scoring through calibration sessions using benchmark scripts.

#### 3.3.2 Digital Critical Thinking Scale (DCTS)

Digital critical thinking was measured using an adapted Digital Critical Thinking Scale (DCTS) consisting of 20 Likert-type items (1–5), yielding total scores ranging from 20 to 100. The scale comprises four subscales corresponding to distinct dimensions of digital critical thinking and AI literacy relevant to generative AI use contexts. Item D17 was reverse-coded so that higher scores consistently reflect stronger digital critical thinking/AI literacy.

To establish instrument reliability, Cronbach’s alpha was computed for the total scale and all subscales at both measurement points. Internal consistency for the total scale was very high at both time points (*α* = 0.97 pretest; α = 0.99 posttest), with subscale reliability estimates ranging from 0.77 to 0.97 at pretest and 0.94–0.98 at posttest. These values exceed the conventionally accepted threshold of α = 0.70, supporting the scale’s reliability for the present sample. However, given the modest sample size (*N* = 53), these estimates should be interpreted cautiously as preliminary indicators of internal consistency pending replication with larger samples.

#### 3.3.3 Qualitative reflection instrument

A brief background questionnaire recorded gender, age, major, self-reported English proficiency, and prior experience with AI tools. To capture process evidence and responsible-use practices, the experimental group completed a structured reflection sheet and an open-ended prompt at the intervention’s conclusion, focusing on AI-supported writing routines, verification behaviors, and responsible-use regulation. These data served as the primary source for thematic analysis and qualitative strand triangulation.

### 3.4 Intervention and control conditions

#### 3.4.1 Experimental condition: AI-supported writing instruction

Both groups followed the same syllabus, course objectives, and assessment schedule throughout the instructional period. The experimental group additionally received explicit instruction in a guided human–AI collaboration workflow built around five interrelated components: problem framing and prompt design; generating alternative outlines, arguments, and claims; iterative drafting and revision aligned with rhetorical purpose and academic conventions; verification and triangulation of factual claims and citations using scholarly databases and institutional resources; and responsible-use regulation, encompassing attribution decisions, management of AI reliance, and maintenance of authorial ownership. Students used a generative AI assistant primarily for idea development and structural scaffolding, alongside a language-feedback tool for editing and surface-level accuracy improvement. Each major assignment included an AI-use log in which students documented prompts submitted, outputs consulted, decisions to accept or reject AI suggestions, and justifications for those decisions—a design feature intended to sustain learner agency and cultivate critical evaluation habits throughout the writing process.

#### 3.4.2 Control condition: traditional writing instruction

The control group completed the same writing tasks and received equivalent instructor feedback and peer review opportunities, but did not receive systematic training in AI-supported workflows and did not use AI tools as a structured component of guided coursework. Any incidental AI use by control-group students was neither incorporated into instruction nor mediated through course activities, and therefore did not constitute the kind of scaffolded, metacognitively guided engagement that characterized the experimental condition.

### 3.5 Procedure

The intervention was implemented over 11 weeks as follows:

- Week 1: Study orientation, informed consent, pretest writing task administration, and pretest DCTS completion.
- Week 2: Introduction to human–AI collaboration principles; problem framing and prompt design training.
- Week 3: Generating alternative outlines and argumentative structures using the generative AI assistant.
- Week 4: First guided writing assignment with AI-use log documentation.
- Week 5: Iterative drafting and revision cycles; comparing AI-generated alternatives against student drafts.
- Week 6: Verification and triangulation training; cross-checking AI claims using Google Scholar and institutional databases.
- Week 7: Second guided writing assignment incorporating verification routines.
- Week 8: Responsible-use regulation instruction; attribution decisions and authorship maintenance.
- Week 9: Third guided writing assignment with full human–AI collaboration workflow.
- Week 10: Peer review, reflective revision, and AI-use log review.
- Week 11: Posttest writing task, posttest DCTS administration, and submission of structured reflection sheets.

### 3.6 Data analysis

Quantitative data were screened for completeness and plausibility; only participants with complete pre/post measurements were retained. Baseline equivalence was assessed using independent-samples *t*-tests for continuous variables and Chi-square or Fisher’s exact tests for categorical variables. Primary intervention effects were evaluated using independent-samples *t*-tests comparing posttest outcomes between groups; within-group change was reported descriptively using paired-samples *t*-tests. Gain-score comparisons (post minus pre) were reported as Supplementary file 1. Effect sizes are reported as Cohen’s dz. for within-group change and Hedges’ *g* for between-group differences, with 95% confidence intervals. Associations between writing and DCTS outcomes were examined using Pearson correlations with 95% confidence intervals. Although baseline equivalence was confirmed at pretest, independent-samples *t*-tests on posttest scores do not statistically control for residual pretest variability. ANCOVA would provide more precise estimates; however, given confirmed baseline equivalence and small sample size, gain-score analyses are reported as converging supplementary evidence, and results should be interpreted as descriptive between-group comparisons rather than covariate-adjusted causal estimates.

Because participants were assigned by intact course sections rather than individual randomization, observations within each section may not be fully independent, introducing potential intraclass clustering. Standard independent-samples *t*-tests do not account for this clustering, and as a result, standard errors may be underestimated and *p*-values optimistic. A clustering-aware approach such as multilevel modeling or cluster-robust standard errors would be more appropriate; however, with only two clusters, such models are not estimable in the present data. Accordingly, all inferential results should be interpreted with caution as preliminary estimates rather than definitive causal evidence. Qualitative responses were analyzed thematically using an iterative three-stage procedure: open coding of all excerpts, consolidation of codes into categories, and refinement of themes through constant comparison. NVivo software supported systematic organization and retrieval of qualitative data. Quantitative and qualitative strands were integrated through triangulation and a joint display linking outcome patterns to process evidence from learner reflections.

## 4 Results

### 4.1 Descriptive statistics and baseline equivalence

Descriptive statistics are presented first to characterize the distribution of scores across groups and time points, followed by inferential analyses to evaluate intervention effects and between-group differences. Table 1 presents descriptive statistics for writing proficiency and DCTS total by group at pretest and posttest, together with gain scores. Baseline equivalence analyses showed no statistically significant pretest differences between the experimental and control groups for writing proficiency, *t*(51) = 0.03, *p* = 0.977, or DCTS total, *t*(51) = 0.33, *p* = 0.741. Categorical background variables were also comparable across groups (Chi-square/Fisher tests, *p*s ≥ 0.90), indicating that observed post-intervention differences are unlikely to reflect substantial pre-existing group disparities.

**Table 1:** Descriptive statistics for writing proficiency and DCTS by group (pretest, posttest, and gain).

Measure	Group	Pretest M (SD)	Posttest M (SD)	Gain M (SD)
Writing proficiency (0–20)	Experimental (*n* = 31)	11.19 (1.51)	15.23 (1.65)	4.03 (0.48)
Writing proficiency (0–20)	Control (*n* = 22)	11.18 (1.40)	11.91 (1.44)	0.73 (0.46)
DCTS total (20–100)	Experimental (*n* = 31)	57.03 (10.20)	89.84 (10.25)	32.81 (6.62)
DCTS total (20–100)	Control (*n* = 22)	56.14 (8.88)	56.18 (8.94)	0.05 (0.21)

Gain = Posttest − Pretest. Writing proficiency scores range from 0 to 20. DCTS total ranges from 20 to 100; item D17 was reverse-coded prior to scoring. SD, standard deviation.

### 4.2 Intervention effects on writing proficiency and DCTS

Within-group analyses indicated substantial improvement in the experimental group from pretest to posttest on writing proficiency, *t*(30) = 46.58, *p* < 0.001. The control group also showed a statistically significant increase, *t*(21) = 7.48, *p* < 0.001; however, the magnitude of change was negligible in practical terms (gain *M* = 0.73 on a 20-point scale). Between-group comparisons at posttest indicated a large and highly significant advantage for the experimental group, *t*(51) = 7.60, *p* < 0.001, with a mean difference of 3.32 points (95% CI [2.44, 4.20]) and a large standardized effect (Hedges’ *g* = 2.09, 95% CI [1.41, 2.76]). For DCTS total, the experimental group showed large gains from pretest to posttest, *t*(30) = 27.59, *p* < 0.001, whereas the control group showed no meaningful change, *t*(21) = 1.00, *p* = 0.329. Between-group comparisons confirmed a very large advantage for the experimental group at posttest, *t*(51) = 12.41, *p* < 0.001, with a mean difference of 33.66 DCTS points (95% CI [28.21, 39.11]) and a very large standardized effect (Hedges’ *g* = 3.41, 95% CI [2.55, 4.26]). Table 2 summarizes the between-group effect-size estimates for both primary outcomes.

**Table 2:** Between-group posttest comparisons: writing proficiency and DCTS.

Outcome	Exp. M (SD)	Ctrl. M (SD)	Mean Diff. (95% CI)	Hedges’ *g* (95% CI)	*p*
Writing proficiency	15.23 (1.65)	11.91 (1.44)	3.32 [2.44, 4.20]	2.09 [1.41, 2.76]	<0.001
DCTS total	89.84 (10.25)	56.18 (8.94)	33.66 [28.21, 39.11]	3.41 [2.55, 4.26]	<0.001

Exp. = Experimental group (*n* = 31); Ctrl. = Control group (*n* = 22). Mean Diff. = Experimental minus control posttest mean. Hedges’ *g* is reported with 95% confidence intervals. DCTS, Digital Critical Thinking Scale.

**Figure 1:**
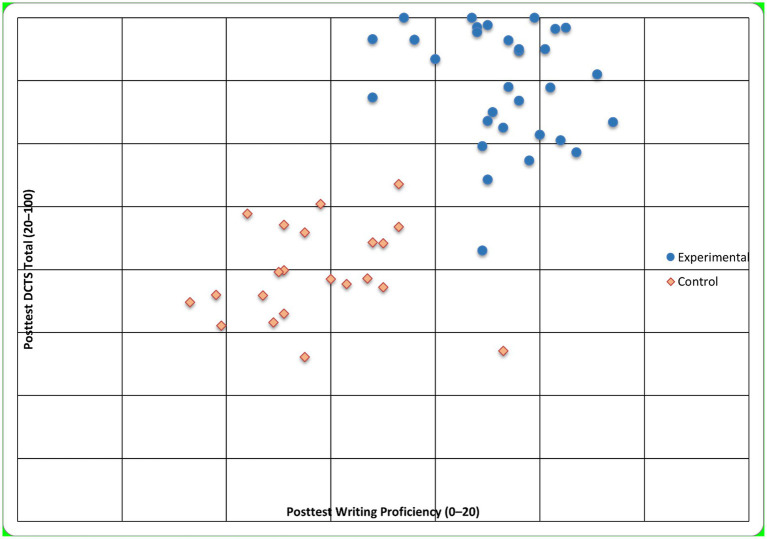
Posttest writing proficiency versus DCTS scores.

Figure 1 presents a scatter plot of posttest writing proficiency against posttest DCTS scores for all participants (*N* = 53), visually confirming the strong positive association (*r* = 0.94) reported in Table 3.

**Table 3:** Pearson correlations between writing proficiency and DCTS (*N* = 53).

Variable pair	*r*	95% CI	*p*
Pretest writing × Pretest DCTS	0.81	[0.69, 0.89]	<0.001
Posttest writing × Posttest DCTS	0.94	[0.90, 0.97]	<0.001
writing gain × dcts gain	0.93	[0.88, 0.96]	<0.001

All *p*-values are < 0.001. DCTS = Digital Critical Thinking Scale total score. Gain = Posttest minus pretest score.

### 4.3 Associations between writing proficiency and digital critical thinking

Pearson correlations indicated strong positive associations between writing proficiency and DCTS in the full sample (*N* = 53) at all measurement points. The relationship was substantial at pretest (*r* = 0.81, 95% CI [0.69, 0.89], *p* < 0.001) and strengthened markedly at posttest (*r* = 0.94, 95% CI [0.90, 0.97], *p* < 0.001). Gain-score correlations were similarly strong (*r* = 0.93, 95% CI [0.88, 0.96], *p* < 0.001), indicating that participants who improved more in digital critical thinking tended to also improve more in writing proficiency across the instructional interval. Table 3 presents the full correlation matrix. Because both outcomes were influenced by group assignment and were measured over the same intervention interval, these associations are interpreted as descriptive co-development patterns rather than evidence of a unidirectional causal mechanism.

### 4.4 Learner reflections: quantitative indicators

Structured reflection items from the experimental group (*n* = 31) indicated that students perceived AI as helpful for improving both organizational quality and language accuracy, while reporting relatively moderate levels of reliance on AI output and high rates of output verification prior to incorporation into their writing. Table 4 presents descriptive data from the structured reflection instrument.

**Table 4:** Experimental-group structured reflection item responses (*n* = 31).

Reflection item	M (SD)	Scale
AI improved content/organization of my writing	3.48 (0.68)	1–4
AI improved language accuracy and style	3.39 (0.50)	1–4
I relied on AI beyond course guidance	2.10 (0.30)	1–3
I verified AI outputs before using them	4.00 (0.86)	1–5

Higher values indicate stronger agreement or greater frequency. Scale ranges vary by item as indicated.

### 4.5 Qualitative thematic analysis: emergent themes

Thematic analysis of open-ended experimental-group reflections yielded four interrelated themes that collectively characterize students’ engagement with AI during the intervention period. Table 5 presents the themes with associated codes, illustrative excerpts, and frequency information. All four themes were evident across all or nearly all participant reflections, reflecting the consistency of students’ self-reported AI-engagement practices.

**Table 5:** Qualitative themes, key codes, and illustrative excerpts (experimental group, *n* = 31).

Theme	Key codes	Illustrative excerpt	Freq.
1. AI as a flexible writing scaffold	Idea generation; outlining; lexical support; genre framing	“It gave me a clear outline with three advantages; I kept the structure but changed one point to fit my own argument.”	100%
2. Detecting and repairing AI errors	Hallucinated citations; misleading statistics; cultural mismatch; tone mismatch	“It gave a fake citation for Al-Ghamdi (2023), so I removed it and looked for a real study before adding a reference.”	100%
3. Verification as digital critical practice	Cross-checking via Google Scholar; textbook; web search; plausibility checks	“I cross-checked the information with Google Scholar and our textbook, and I only kept what matched reliable sources.”	81%
4. Metacognitive regulation and ethical positioning	Managing reliance; staged use; time limits; rules of engagement; integrity awareness	“Next time I’ll write the first draft without AI and only use it to revise and check sources so the final essay is my own.”	100%

Freq. = percentage of participants whose reflections contained evidence of this theme. Excerpts are presented verbatim as submitted by participants.

Theme 1, AI as a flexible writing scaffold, captured students’ consistent framing of AI tools as supports for their own thinking and composing process rather than substitutes for authorship. Students described using AI to generate ideas and organize arguments through requesting outlines and lists of alternative claims, and then selectively adapting those suggestions to fit their own argumentative stance. Lexical and sentence-level refinement was also prominent, including requests for more precise academic vocabulary, correction of grammatical errors, simplification of complex sentences, and improvement of academic register. Crucially, students typically adopted structural or lexical guidance while rewriting content in their own words and anchoring claims in course readings, consistently emphasizing ownership of the final product.

Theme 2, detecting and repairing inaccurate or inappropriate AI output, captured how students recognized and actively responded to AI limitations rather than accepting outputs uncritically. A prominent code involved hallucinated or fabricated information, especially citations to non-existent studies and unverifiable statistical claims. Students also identified cultural and contextual mismatches—cases where AI offered examples unsuited to the Saudi educational context or made sweeping generalizations that required qualification. A third code related to tone and lexical register, with students identifying when AI language was overly complex, dramatic, or insufficiently academic, and revising or rejecting such content accordingly. Theme 2 thus portrays students as active editors and correctors who scrutinized AI output for factual reliability, contextual fit, and academic appropriateness.

Theme 3, verification as a routine digital critical thinking practice, documented how students evaluated the reliability of AI-generated content through systematic triangulation with external sources rather than treating AI outputs as inherently authoritative. Students described cross-checking AI claims via Google Scholar, library databases, reputable news outlets, and official websites, retaining only content that could be externally supported. Others verified AI suggestions against course-based materials including the textbook, assigned articles, and institutional policies on acceptable AI use. Plausibility checks and peer or instructor consultation also featured prominently as verification strategies. These behaviors indicate that verification became a habitual practice rather than an occasional corrective step, directly reflecting the DCTS emphasis on evaluation and source triangulation.

Theme 4, metacognitive regulation and ethical self-positioning, captured how students monitored and reflected on their reliance on AI and articulated concrete strategies for more controlled future use. Most students described their reliance as appropriate, using metaphors positioning AI as ‘a tool, not a perfect human’ or ‘a helpful friend.’ Looking forward, students outlined specific self-regulation strategies: drafting independently before using AI, restricting AI to defined functions such as grammar checks and vocabulary refinement, setting time limits on AI interaction, and verifying sources immediately rather than near submission. Ethical positioning was explicit throughout this theme, with students emphasizing maintenance of authorship, avoidance of plagiarism, and alignment with course expectations as central concerns governing their AI use decisions.

### 4.6 Integration of quantitative and qualitative evidence

Table 6 presents the joint display integrating quantitative outcomes with qualitative themes to support meta-inferences about mechanisms and conditions. The strongest quantitative improvements—in writing performance and digital critical thinking/AI literacy—align closely with qualitative evidence of deliberate, responsibility-oriented engagement with AI: planning and revision scaffolding from Theme 1 supports the writing proficiency gains; error detection from Theme 2 aligns with DCTS improvements; triangulation behaviors from Theme 3 explain the strengthening correlation between the two outcomes; and the ethical self-regulation of Theme 4 supports the conditional theoretical claim that structured, responsibility-oriented AI use is the proximate mechanism for beneficial dual outcomes. The contrast with the control condition—which showed minimal writing gain and negligible DCTS change without any parallel process evidence—reinforces the interpretation that guided AI routines, rather than general course participation, account for the observed outcome pattern.

**Table 6:** Joint display integrating quantitative results and qualitative themes.

Quantitative finding	Qualitative theme and codes	Illustrative excerpt	Integrated meta-inference
Writing: Post MExp = 15.23 vs. MCtrl = 11.91; Hedges’ *g* = 2.09; raw gain 4.03 vs. 0.73	Theme 1 (100%): AI as scaffold for planning, structure, lexical support	“I kept the structure but changed one point and wrote the examples in my own words.”	Writing gains align with AI functioning as a planning/revision scaffold while students retain authorship decisions.
DCTS: Post MExp = 89.84 vs. MCtrl = 56.18; Hedges’ *g* = 3.41; raw gain 32.81 vs. 0.05	Theme 2 (100%): Detecting/correcting hallucinated or misfitting outputs	“It gave a fake citation. So I removed it and searched for a real study.”	DCTS gains align with students treating AI outputs as claims to be tested, not authoritative answers.
Strong posttest linkage: *r* = 0.94; gain-score linkage: *r* = 0.93	Theme 3 (81%): Triangulation with credible sources and course materials	“I only kept what matched reliable sources.”	Co-development suggests shared practices: verification and revision support both credible writing and digital critical thinking.
DCTS reliability: alpha = 0.97 (pre), 0.99 (post)	Theme 4 (100%): Rules of engagement, staged AI use, integrity regulation	“First draft without AI. use it to revise and check sources.”	Responsibility-oriented self-regulation appears to be a key condition for beneficial AI mediation.

Exp., Experimental group; Ctrl., Control group. Hedges’ *g* values are for between-group posttest comparisons. Qualitative frequency percentages refer to the proportion of experimental-group participants whose reflections contained evidence of each theme.

## 5 Discussion

This study examined whether structured AI-supported writing instruction could enhance EFL students’ writing proficiency and digital critical thinking, and whether gains in both domains would co-develop systematically. Using a convergent mixed-methods design grounded in constructivist–sociocultural theory, the study compared an AI-enriched writing course with traditional instruction. Findings converge on one overarching conclusion: guided, responsibility-oriented human–AI collaboration produced substantial gains in writing and marked improvements in digital critical thinking, with all three null hypotheses rejected. Crucially, the intervention did not merely elevate test scores; it reshaped how students composed and critically evaluated text within AI-rich academic environments (Deep and Chen, 2025; Aljuaid, 2024; Urmeneta and Romero, 2025).

The experimental group’s substantially larger writing gains—from virtually identical pretest baselines—corroborate evidence that AI tools improve EFL writing most effectively when embedded within explicit pedagogical frameworks (Al-Mahmud, 2023; Abduljawad, 2024; Jamshed et al., 2024). Systematic reviews confirm that benefits are strongest within process-writing pedagogy emphasizing iterative drafting and reflective revision, not standalone productivity use (Aljuaid, 2024; Deep and Chen, 2025). Qualitative findings provide the mechanistic account: students used AI as a scaffold for ideation, outlining, and lexical refinement while maintaining authorial control through selective uptake and source-anchored rewriting—a pattern aligning precisely with sociocultural accounts of mediated learning within zones of proximal development (Liu et al., 2025; Hashemifardnia and Kooti, 2025).

The very large between-group DCTS difference supports the core AI-literacy claim that digital critical thinking must be an explicit instructional target, not an assumed by-product of technology use (Alotaibi and Alshehri, 2023; Zakaria et al., 2025). Qualitative themes directly mapped onto DCTS dimensions: Theme 2 showed students treating AI outputs as provisional claims requiring verification; Theme 3 documented systematic triangulation with credible sources; Theme 4 revealed metacognitive monitoring and integrity-boundary articulation. These enacted behaviors lend construct validity to the quantitative findings and offer a coherent mechanistic explanation for the large DCTS gains observed exclusively in the experimental condition (Faisal, 2024; Elmahdi et al., 2025).

However, the exceptionally large effect sizes warrant critical scrutiny. Several factors beyond genuine instructional effectiveness may have contributed to their magnitude. First, novelty effects are plausible: heightened engagement with unfamiliar AI tools may have temporarily inflated performance and self-reported scores independently of durable skill gains. Second, instructional intensity likely played a role, as the experimental condition involved substantially more structured cognitive activity than the control condition, meaning differences may partly reflect differential engagement rather than AI tool effects specifically. Third, the DCTS, administered to a group that had explicitly practiced the behaviors it measures, may have been particularly responsive to intervention content, potentially overstating gains. Finally, high posttest alpha values may reflect item redundancy rather than broad construct coverage. The direction of findings is credible, but effect magnitudes should be interpreted as preliminary upper-bound estimates pending replication with larger randomized samples.

The strengthening correlation between writing proficiency and digital critical thinking—from pretest (*r* = 0.81) to posttest (*r* = 0.94), with gain scores also strongly associated (*r* = 0.93)—provides preliminary evidence that these are co-developing competencies rather than independent outcomes (Zakaria et al., 2025; Deep and Chen, 2025). The same habits used to interrogate AI outputs—rejecting unsupported claims, verifying sources, grounding evidence contextually—also shaped substantive writing decisions and improved essay coherence and evidentiary quality. This pattern is consistent with frameworks positioning critical thinking as an enabling condition for advanced academic writing, suggesting the intervention fostered integrated competency development rather than isolated gains in parallel skill domains (Al-Zahrani and Alasmari, 2025; Elmahdi et al., 2025).

The intervention activated a coupled developmental pathway across three interrelated dimensions. First, AI-supported workflows leveraged creativity-relevant functions—brainstorming, alternative outlining, argument reframing—without delegating authorship or bypassing cognitive demands. Second, cognitive intelligence was operationalized as digital critical thinking under informational uncertainty: evaluating credibility, verifying claims, and justifying inclusion decisions. Third, socio-emotional and ethical growth appeared in students’ metacognitive self-monitoring and explicit personal rules governing reliance, time use, and attribution integrity. This triadic alignment explains why writing and DCTS improvements were parallel and tightly linked, and why students described AI simultaneously as a composing scaffold and an object of critical scrutiny (Habib et al., 2024; Urmeneta and Romero, 2025; Garzón et al., 2025).

The findings align with Vision 2030 arguments for ethically grounded AI integration in Saudi higher education (Alotaibi and Alshehri, 2023; Al-Zahrani and Alasmari, 2025). Rather than framing generative AI as an integrity threat or a mere productivity shortcut, the intervention demonstrates a feasible instructional posture: AI as cognitive scaffold and critical sparring partner, governed through explicit responsibility routines. This directly addresses Saudi scholarship concerns about overreliance and erosion of independent thinking (Al-Sofi, 2024; Al-Harbi and Al-Ahdal, 2025; Al-Nofaie and Alwerthan, 2024), showing that such risks are pedagogically manageable through design—not prohibition—while honoring institutional integrity expectations and cultural values of ethical academic practice.

The present findings contrast with concerns frequently raised regarding unstructured AI use. Prior research consistently identifies three risks: over-reliance, whereby students delegate composing decisions rather than developing independent judgment (Al-Sofi, 2024; Al-Nofaie and Alwerthan, 2024); superficial learning, whereby AI-generated text bypasses the cognitive demands of planning and revision central to writing development (Deep and Chen, 2025; Aljuaid, 2024); and reduced authorship control, whereby students report diminished ownership of AI-assisted texts (Alyami et al., 2025; Al-Harbi and Al-Ahdal, 2025). The present intervention addressed all three through agency-preserving design features: AI-use logs preserved authorial decision-making; verification routines countered superficial acceptance; and ethical self-regulation instruction targeted over-reliance. Students’ reported moderate reliance, high verification rates, and explicit authorship maintenance suggest these features successfully mitigated risks commonly associated with unstructured generative AI use.

Equally important are the boundary conditions under which AI-supported instruction may be less effective or counterproductive. First, low scaffolding represents a critical threshold: without structured workflows and accountability mechanisms, risks of over-reliance and superficial learning are substantially elevated. Second, students with weak digital critical thinking or limited metacognitive regulation may lack the evaluative capacities needed to interrogate outputs and detect hallucinations—conditions under which AI may reinforce rather than remediate skill deficits. Third, overreliance is a dynamic risk: even within structured interventions, progressive delegation of composing decisions may gradually erode independent authorial judgment in ways not immediately detectable through posttest measures. Finally, limited institutional support—including unclear AI-use policies and insufficient instructor training—may undermine the ethical self-regulation behaviors central to beneficial outcomes here. The instructional model is therefore contingent on scaffolding quality, metacognitive readiness, and institutional governance.

Three practical implications emerge. First, AI should be embedded within guided workflows requiring students to frame problems, draft iteratively, and log accept/reject/justify decisions—not used as unstructured open access (Aljuaid, 2024; Al-Fraidan, 2024). Second, digital critical thinking and AI literacy should be explicitly taught and assessed through dedicated instruments, not assumed as incidental outcomes of technology exposure (Zakaria et al., 2025; Alotaibi and Alshehri, 2023). Third, institutional standards on authorship, attribution, and academically appropriate AI use must be integrated into instruction, equipping students to exercise both technical judgment and ethical discernment when navigating the blurred boundaries of AI-assisted academic writing (Faisal, 2024; Elmahdi et al., 2025).

Several limitations warrant consideration. First, group assignment used intact sections within a single institution, meaning observations may not be fully independent due to intraclass clustering. Standard *t*-tests do not account for this clustering, potentially producing optimistic *p*-values; with only two clusters, multilevel modeling was not estimable, and results should be interpreted as preliminary. The modest sample (*N* = 53) from a single institution limits external validity; findings should not be generalized beyond comparable Saudi EFL contexts without replication across multiple institutions and diverse populations. Third, writing was assessed via one task per time point without reported inter-rater reliability or blinded scoring. Fourth, DCTS relied entirely on self-report, and high alpha values may reflect item redundancy rather than construct breadth; with *N* = 53, reliability estimates should likewise be treated as preliminary, as smaller samples produce less stable coefficients. Finally, qualitative reflections were collected only from the experimental group, preventing direct process-level comparison with the control condition. Future studies should employ randomized designs, multilevel modeling, covariate-adjusted analyses—including ANCOVA with pretest covariates—multiple writing prompts, and process data collected systematically across both conditions to strengthen causal inference and confirm instrument reliability.

## 6 Conclusion

This study provides preliminary quasi-experimental evidence that carefully designed, agency-preserving human–AI collaboration can develop both writing proficiency and digital critical thinking concurrently in authentic EFL university classrooms. By coupling ideation and revision support with verification routines, hallucination-detection instruction, and structured ethical self-regulation, the AI-supported condition produced large, co-related gains in both outcomes—suggesting integrated competency development rather than isolated skill improvement. Rather than treating generative AI as a shortcut or integrity threat, the findings support an instructional model positioning AI as a governed scaffold for critical, responsible academic composing. Future research should test this pathway across disciplines, tool types, and proficiency levels, incorporating measures of creativity, writing self-efficacy, and ethical sensitivity alongside the core outcomes examined here.

## Data Availability

The original contributions presented in the study are included in the article/Supplementary material, further inquiries can be directed to the corresponding author.
