# Supplementary material for: From tool to scaffold: structured human–AI collaboration and its effects on academic writing and digital critical thinking among Saudi EFL learners
Source: Front Psychol. 2026 May 26;17:1830103. doi: 10.3389/fpsyg.2026.1830103 (PMC13249262; doi:10.3389/fpsyg.2026.1830103)
Supplement: Supplementary file 1 [file Supplementary_file_1.DOCX]

**Appendix: Digital Critical Thinking Scale (DCTS) Items**

Participants responded to the following 20 items on a 5-point Likert scale (1 = Strongly Disagree, 5 = Strongly Agree).

D1: I can distinguish between reliable and unreliable digital information.

D2: I evaluate the credibility of online sources before using them.

D3: I verify AI-generated content against trusted academic sources.

D4: I can identify biased or misleading information in digital environments.

D5: I critically assess the accuracy of AI-generated claims.

D6: I cross-check information from multiple sources before accepting it.

D7: I recognize when AI tools produce hallucinated or fabricated content.

D8: I apply critical thinking when interacting with AI-generated outputs.

D9: I evaluate the relevance of digital information to my academic tasks.

D10: I can detect logical inconsistencies in AI-generated text.

D11: I assess the ethical implications of using AI in academic writing.

D12: I make informed decisions about acceptable levels of AI assistance.

D13: I maintain authorial ownership when using AI writing tools.

D14: I document and justify my decisions when accepting or rejecting AI suggestions.

D15: I use AI tools responsibly in accordance with academic integrity standards.

D16: I reflect on my reliance on AI tools during the writing process.

D17: I accept AI-generated content without verifying its accuracy. (Reverse-coded)

D18: I triangulate AI-generated information with scholarly databases.

D19: I regulate my use of AI tools to preserve independent thinking.

D20: I apply ethical reasoning when making attribution decisions in AI-assisted writing.
